# Impact of adequate empirical combination therapy on mortality from bacteremic *Pseudomonas aeruginosa* pneumonia

**DOI:** 10.1186/1471-2334-12-308

**Published:** 2012-11-16

**Authors:** So-Youn Park, Hyun Jung Park, Song Mi Moon, Ki-Ho Park, Yong Pil Chong, Mi-Na Kim, Sung-Han Kim, Sang-Oh Lee, Yang Soo Kim, Jun Hee Woo, Sang-Ho Choi

**Affiliations:** 1 Departments of Infectious Diseases, Asan Medical Center, University of Ulsan College of Medicine, 388-1 Pungnap-dong, Songpa-gu, Seoul, Republic of Korea; 2 Departments of Laboratory Medicine, Asan Medical Center, University of Ulsan College of Medicine, 388-1 Pungnap-dong, Songpa-gu, Seoul, Republic of Korea

**Keywords:** *Pseudomonas aeruginosa*, Pneumonia, Combination treatment

## Abstract

**Background:**

*Pseudomonas aeruginosa* has gained an increasing amount of attention in the treatment of patients with pneumonia. However, the benefit of empirical combination therapy for pneumonia remains unclear. We evaluated the effects of adequate empirical combination therapy and multidrug-resistance in bacteremic *Pseudomonas* pneumonia on the mortality.

**Methods:**

A retrospective cohort study was performed at the 2,700-bed tertiary care university hospital. We reviewed the medical records of patients with bacteremic pneumonia between January 1997 and February 2011. Patients who received either inappropriate or appropriate empirical therapy were compared by using marginal structural model. Furthermore, we investigated the direct impact of combination therapy on clinical outcomes in patients with monomicrobial bacteremic pneumonia.

**Results:**

Among 100 consecutive patients with bacteremic *Pseudomonas* pneumonia, 65 patients were classified in the adequate empirical therapy group, 32 of whom received monotherapy and 33 combination therapy. In the marginal structural model, only inadequate therapy was significantly associated with 28-day mortality (*p =* 0.02), and multidrug-resistance was not a significant risk factor.

To examine further the direct impact of combination therapy, we performed a subgroup analysis of the 65 patients who received adequate therapy. Multivariate logistic regression analysis identified absence of septic shock at the time of bacteremia (OR, 0.07; 95% CI, 0.01-0.49; *p =* 0.008), and adequate combination therapy (OR, 0.05; 95% CI, 0.01-0.34; *p =* 0.002) as variables independently associated with decreased all-cause 28-day mortality.

**Conclusions:**

Our study suggests that adequate empirical combination therapy can decrease mortality in patients with bacteremic *Pseudomonas* pneumonia.

## Background

*Pseudomonas aeruginosa* has emerged as a significant nosocomial pathogen with a substantial frequency of multidrug resistance (MDR) [1]. Moreover pneumonia caused by *P. aeruginosa* is known to be associated with greater mortality than infection of other sites [2-5]. Hence, treatment of *P. aeruginosa* pneumonia poses a great challenge to clinicians. Early studies, mainly of neutropenic patients with bacteremia, showed that mortality was decreased in patients receiving combination therapy [6]. However, the results of subsequent clinical studies on the effect of combination therapy on the treatment of severe *P. aeruginosa* infection have been conflicting [7-11]. These previous studies had significant limitations, including the enrollment of only small numbers of patients infected with different kinds of gram-negative bacilli and the lack of controls. Furthermore, the validity of diagnoses of *P. aeruginosa* pneumonia has itself been questioned because *P. aeruginosa* often colonizes the respiratory tract in hospital settings, and it is often difficult for clinicians to distinguish a colonizer from a true pathogen [2]. Thus, the isolation of *P. aeruginosa* from blood cultures, as well as respiratory specimens, constitutes strong evidence of true *Pseudomonas* pneumonia.

To help identify the best strategy for antibiotic therapy in *Pseudomonas* pneumonia, we evaluated the impact of empirical combination therapy on mortality in 100 consecutive patients with bacteremic *P. aeruginosa* pneumonia using a risk stratification model to adjust for potential differences.

## Methods

### Data collection

All patients ≥18 years with *P. aeruginosa* bacteremic pneumonia between January 1997 and February 2011 were included. Patients with blood cultures positive for *P. aeruginosa* were identified from the computerized database of the clinical microbiology unit. Infectious diseases doctors then reviewed the medical records of these patients and collected demographic, clinical, and microbiological data. Ultimately, only patients with *Pseudomonas* pneumonia (see below) were included in the analysis. Patients with polymicrobial infections were excluded. The study was approved by the Asan Medical Center Institutional Review Board (S2012-1034-0001), and the requirement for patient consent was waved due to the retrospective nature of the study.

### Definitions

Bacteremia was defined as ≥ 1 positive blood culture for *P. aeruginosa* and the presence of the clinical features compatible with infection. If a patient had undergone recurrent episodes of *P. aeruginosa* bacteremia during the study period, only the first episode was considered. Pneumonia was defined as presence of: (1) new radiographic infiltration; (2) one or more of the following symptoms consistent with pneumonia (fever, cough, pleuritic chest pain, and dyspnea); and (3) isolation of *P. aeruginosa* from cultures of bronchoalveolar lavage (BAL) fluid or appropriate respiratory specimens [12,13]. The categories of pneumonia have been previously defined [14,15]. Empirical antibiotic therapy was considered adequate if therapy given intravenously within 48 h of the onset of pneumonia included antimicrobials to which the isolate was susceptible. Patients who received adequate treatment were stratified into 2 groups: a monotherapy group that received only 1 active antimicrobial, and a combination therapy group that simultaneously received 2 active antimicrobials [16]. Antimicrobial susceptibility testing was performed using the MicroScan system with the Neg Breakpoint Combo Panel 44 (Siemens Healthcare Diagnostics Inc., West Sacramento, CA) according to standard criteria of the Clinical and Laboratory Standards Institute [17]. MDR was defined as resistance to more than three classes of antibiotics such as anti-pseudomonal beta-lactams, carbapenems, fluoroquinolones, and aminoglycosides [18].

### Statistical analysis

All statistical analyses were performed using SAS software, version 9.1 (SAS Institute Inc, Cary, NC). Marginal structural models were used to estimate the difference in mortality between the adequate therapy group and the inadequate therapy group. Careful adjustment was made using weighted Cox proportional-hazards regression models that were based on the inverse-probability-of-treatment weighted (IPTW) method in order to reduce the effect of potential confounding factors and selection bias in this observational study [19]. IPTW estimation relies on multivariable logistic regression analysis. All variables with a p value < 0.2 on univariable analysis were then introduced into the multivariable logistic regression model, and a backwards stepwise logistic regression was carried out. Sex, age, underlying diseases, multidrug resistance, McCabe score, APACHE II score, Pitt bacteremia score, clinical pulmonary infection score (CPIS), type of pneumonia, and use of previous antibiotics were classified as independent variables for the purposes of this study. P value ≤ 0.05 was considered statistically significant.

## Results

During the study period 1,361 patients with *P. aeruginosa* bacteremia were identified from our hospital records. One hundred (7.3%) patients with concomitant pneumonia were eventually included in the analysis. The demographics, clinical features, microbiologic characteristics and clinical outcomes of these patients are shown in Table 1. Hematologic malignancy (34%) was the most common underlying disease, followed by solid cancer (23%). Of the 100 cases, 37 (37%) originated in the community (18 community-acquired and 19 healthcare-associated infections). Thirty-eight (38.0%) patients presented with septic shock. Sixty-five patients (65.0%) were assigned to the adequate empirical therapy group, 32 (49.2%) of whom received monotherapy and 33 (50.8%) combination therapy. The antimicrobials used in monotherapy were as follows: ceftazidime (n = 10), cefepime (n = 2), carbapenem (n = 8), piperacillin-tazobactam (n = 7), and ciprofloxacin (n = 5). The remainder of the patients received combination therapy: piperacillin-tazobactam plus ciprofloxacin (n = 11), cefoperazone-sulbactam plus aminoglycoside (n = 9), ceftazidime plus aminoglycoside (n = 8), ceftazidime plus ciprofloxacin (n = 3), and carbapenem plus ciprofloxacin (n = 2). MDR pathogens were commonly found in the inadequate therapy group (42.9% [15 of 35] *vs.* 12.3% [8 of 65], *p* < 0.001). Among the 65 patients in the adequate therapy group, there was no significant difference in terms of the emergence of antimicrobial resistance between the monotherapy and combination therapy group (21.9% [7/32] and 12.1% [4/33], respectively) (*p =* 0.29). However, the 2-week bacteria eradication rate (54.5% [18/33] *vs.*18.8% [6/32], *p =* 0.04) and the 4-week eradication rate (54.5% [18/33] *vs.* 28.1% [9/32], *p =* 0.04) were significantly higher in the combination therapy group than the monotherapy group. There were no subsequent episodes of *Pseudomonas* pneumonia after 1 month, but there were 6 subsequent episodes within the first 6 months (2 patients in the inadequate therapy group [5.7%], 1 patient in adequate monotherapy group [3.1%], and 3 patients in adequate combination therapy group [9.1%]). However, there were no deaths due to recurrent *P. aeruginosa* infection, and all patients with subsequent episodes demonstrated full recoveries.

**Table 1:** Baseline characteristics of patients who received adequate antibiotic monotherapy and combination therapy, and inadequate therapy

	**Total (n = 100)**	**Adequate therapy (n = 65)**		**Inadequate therapy (n = 35)**	***P*value**
		**Monotherapy (n = 32)**	**Combination therapy (n = 33)**		
**Age** (median years, range)	59 (53–67)	60	56	61	0.24
**Male gender**	74 (74.0)	27 (84.4)	24 (72.7)	23 (65.7)	0.22
**Underlying disease**					
Hematologic malignancy	34 (34.0)	12 (37.5)	14 (42.4)	8 (22.9)	0.21
Solid organ malignancy	23 (23.0)	8 (25.0)	12 (36.4)	3 (8.6)	0.02
Neurologic disease	14 (14.0)	7 (21.9)	0	7 (20.0)	0.01
Immunosuppression	22 (22.0)	6 (18.8)	10 (30.3)	6 (17.1)	0.13
Structural lung disease	8 (8.0)	2 (6.3)	4 (12.1)	2 (5.9)	0.65
Congestive heart failure	9 (9.0)	2 (6.3)	1 (3.0)	6 (17.1)	0.13
Hemodialysis	10 (10.0)	2 (6.3)	1 (3.0)	7 (20.0)	0.07
Liver cirrhosis	5 (5.0)	1 (3.1)	2 (6.1)	2 (5.7)	0.99
Biliary disease	2 (2.0)	0	0	2 (5.7)	0.33
**McCabe score**					<0.001
Non-fatal	42 (42.0)	13 (40.6)	7 (21.2)	22 (62.9)	
Ultimately fatal	44 (44.0)	18 (56.3)	20 (60.6)	6 (17.1)	
Rapidly fatal	14 (14.0)	1 (3.1)	6 (18.2)	7 (20.0)	
**APACHE II score**, median (IQR)	21 (16–26)	18.5 (13–22)	20 (17–24.5)	23 (14–27)	0.08
**Pitt bacteremia score**, median (IQR)	3 (1–5)	2 (0–4)	3 (2–5)	4 (2–6)	0.05
**CPIS**, median (IQR)	6 (5–7)	6 (5–7)	6 (5–7)	6 (5–7)	0.57
**Type of pneumonia**					0.01
Community-acquired	18 (18.0)	3 (9.4)	11 (33.3)	4 (11.4)	
Healthcare-associated	19 (19.0)	8 (25.0)	6 (18.2)	5 (14.3)	
Hospital- acquired	44 (44.0)	15 (46.9)	15 (45.5)	14 (40.0)	
Ventilator-associated	19 (19.0)	6 (18.8)	1 (3.0)	12 (34.3)	
**Initial manifestation within 24 h**					0.22
Bacteremia without SIRS	6 (6.0)	2 (6.3)	2 (6.1)	2 (5.7)	
Sepsis	35 (35.0)	17 (53.1)	8 (24.2)	10 (28.6)	
Severe sepsis	21 (21.0)	6 (18.8)	7 (21.2)	8 (22.9)	
Septic shock	38 (38.0)	7 (21.9)	16 (48.5)	15 (42.9)	
**MDR-*P. aeruginosa***	23 (23.0)	6 (18.8)	2 (6.1)	15 (42.9)	0.001
**Previous antibiotic therapy**	55 (55.0)	17 (30.9)	11 (20.0)	27 (49.1)	0.009
**Initial antimicrobial administration within 24 h**	94 (94.0)	29 (90.6)	32 (96.9)	33 (94.3)	0.60
**Total duration of therapy, median days** (IQR)	14 (8–21)	15 (9–19)	14 (8–22)	14 (7–21)	0.71
**Total length of hospital stay, median days** (IQR)	37.5 (19–75)	39 (19–94)	28 (13–60)	52 (26–82)	0.10
**Length of hospital stay before bacteremia, median days** (IQR)	12.5 (0–39)	9 (0–29.5)	0 (0–19.5)	29 (9–59)	0.005
**Length of hospital stay after bacteremia,** median days (IQR)	16.5 (7–36.5)	18.5 (10.8-52.5)	16 (6.5-34)	17 (7–33)	0.21
**Overall mortality**					
7-day mortality	21 (21.0)	5 (15.6)	6 (18.2)	10 (28.6)	0.382
14-day mortality	30 (30.0)	8 (25.0)	6 (18.2)	16 (45.7)	0.035
28-day mortality	51 (51.0)	17 (53.1)	10 (30.3)	24 (68.6)	0.01

Data are presented as number (%) unless otherwise specified.

*IQR* = interquartile range**,***CPIS* = Clinical Pulmonary Infection Score, *MDR* = multidrug-resistant.

We identified 14 patients who had coinfection at the time of *Pseudomonas* pneumonia (6 patients in the inadequate therapy group and 8 patients in the adequate therapy group), though the rates of coinfection were not significantly different between the monotherapy group (9.4% [3 of 32]) and the combination therapy group (15.2% [5 of 33], *p =* 0.71). Of these 14 patients, 8 patients died (4 patients in inadequate therapy, 2 patients in monotherapy, and 2 patients in the combination therapy group), but there were no deaths due to coinfection in either group.

The overall all-cause 28-day mortality was 51.0% (51 of 100). The 28-day mortality was significantly higher in the inadequate therapy group than the adequate therapy group (68.6% [24/35] *vs.* 41.5% [27/65], *p =* 0.01). Variables such as sex, age, MDR, McCabe score, APACHE II score, Pitt bacteremia score, CPIS, and the use of previous antibiotics (which were determined to be significant by univariate analysis) were subjected to logistic regression modeling in order to identify the independent risk factors for all-cause mortality. Multivariate analysis indicated that the APACHE II score (adjusted odds ratio [AOR]: 1.08; 95% confidence interval [CI]: 1.02–1.15; *p =* 0.01) and inadequate therapy (AOR: 2.73; 95% CI: 1.11–6.71; *p =* 0.03) were independently associated with all-cause 28-day mortality (Additional file 1: Table S1). The marginal structural model was estimated using multiple logistic regression analysis. To reduce the effects of potential confounding factors and selection bias, additional analyses were performed with covariable adjustment using IPTW. These additional analyses showed that only inadequate therapy was significantly associated with 28-day mortality (AOR: 3.02; 95% CI: 1.15–7.93; *p =* 0.02) and that MDR was not a significant risk factor (data not shown).

To further examine the direct impact of combination therapy, we performed a subgroup analysis of the 65 patients who received adequate therapy. In univariate analysis, variables that were significantly associated with 28-day mortality included underlying hematologic malignancy, Pitt bacteremia score, initial clinical manifestation of bacteremia, and monotherapy. Multivariate logistic regression analysis identified the absence of septic shock at the time of bacteremia (AOR, 0.07; 95% CI, 0.01-0.49; *p =* 0.008), and combination therapy (AOR, 0.05; 95% CI, 0.01-0.34; *p =* 0.002) as variables that were independently associated with decreased all-cause 28-day mortality (Table 2).

**Table 2:** Logistic regression analysis of the risk factors for 28-day mortality in patients with adequate empirical therapy

**Variable**	**Univariate analysis**	***P***	**Multivariate analysis**	***P***
	**OR (95% CI)**		**OR (95% CI)**	
Age	1.00 (0.96-1.04)	.98		
Male gender	0.48 (0.14-1.76)	.27		
Underlying disease				
Solid organ malignancy	1.22 (0.42-3.56)	.71		
Hematologic malignancy	2.33 (0.84-6.46)	.10		
Structural lung disease	0.25 (0.03-2.31)	.22		
Neurologic disease	0.21 (0.02-1.81)	.15		
Congestive heart failure	2.96 (0.26-34.42)	.39		
Hemodialysis	0.69 (0.06-8.05)	.77		
Immunosuppression	0.62 (0.20-1.93)	.41		
McCabe score				
Non-fatal	0.57 (0.09-3.38)	.54		
Ultimately fatal	1.20 (0.24-6.11)	.83		
Rapidly fatal	1.0 (referent)			
APACHE II score	1.04 (0.97-1.11)	.32		
Pitt bacteremia score	1.21 (0.96-1.51)	.10		
CPIS	1.06 (0.73-1.54)	.77		
Type of pneumonia				
Community-acquired	1.00 (0.16-6.26)	.99		
Healthcare-associated	0.74 (1.12-4.73)	.75		
Hospital- acquired	1.02 (0.19-5.37)	.98		
Ventilator-associated	1.0 (referent)			
MDR-*P. aeruginosa*	0.43 (0.08-2.30)	.32		
Previous antibiotic therapy	1.42 (0.53-3.86)	.49		
Initial manifestation within 24 h				
Sepsis	0.36 (0.11-1.17)	.09	0.07 (0.01-0.49)	0.008
Severe sepsis	0.29 (0.07-1.21)	.09	0.13 (0.02-0.89)	0.04
Septic shock	1.0 (referent)			
Type of adequate empirical therapy				
Monotherapy	0.38 (0.14-1.06)	.06	0.05 (0.01-0.34)	0.002
Combination therapy	1.0 (referent)			

*MDR* = multidrug-resistant.

## Discussion

In our present study, we evaluated the impact of empirical combination therapy on clinical outcomes in 100 consecutive patients with monomicrobial bacteremic *Pseudomonas* pneumonia. Adequate empirical combination therapy was associated with significantly lower 28-day mortality.

Despite the high mortality rates in patients with *P. aeruginosa* pneumonia, it is still not clear how best to treat *Pseudomonas* pneumonia. Paul et al., in their meta-analysis of 64 randomized trials, compared the clinical outcomes of beta lactam monotherapy and beta lactam-aminoglycoside combination therapy for the treatment of sepsis in immunocompetent patients [7]. In their study, beta lactam–aminoglycoside combination therapy was not associated with decreased mortality. On the other hand, Safdar and colleagues previously examined the impact of combination therapy on patients with gram-negative bacteremia and found a survival benefit associated with combination therapy in the subgroup of patients with *P. aeruginosa* bacteremia, demonstrating an approximately 50% reduction in mortality (OR: 0.50; 95% CI: 0.32-0.79) [8]. These differing results may have been due to the limitations of the earlier studies, including: (1) the combinations of different pathogens reported; (2) the greatly varying proportion of patients with pneumonia (range: 14%-100%); (3) the use of aminoglycoside as a monotherapy, which is not currently considered an appropriate therapy for *P. aeruginosa* infection; and (4) the possibility of *P. aeruginosa* colonization of the respiratory tract was not excluded. To overcome these limitations, we included a large number of consecutive patients with monomicrobial bacteremic *Pseudomonas* pneumonia in our study.

In our present analyses, adequate empirical therapy was associated with a significant reduction in mortality after 7 days. Analysis of the adequate empirical therapy group revealed that combination therapy was a variable independently associated with decreased 28-day mortality. One possible explanation for the decreased mortality could be a reduced frequency of emergence of antimicrobial resistance. However, there was no significant difference between the emergence of antimicrobial resistance in the monotherapy group (21.9% [7/32]) and the combination therapy group (12.1% [4/33], *p =* 0.29). Even in cases in where antimicrobial resistance emerged, only 3 patients (3 of 7) and 1 patient (1 of 4) died, respectively. Thus, the emergence of resistance may not affect the mortality rate. In contrast, the 2-week (18.8% *vs.* 54.5%, *p =* 0.04) and 4-week bacteria eradication rates (28.1% *vs.* 54.5%, *p =* 0.04) after bacteremia were significantly higher in the combination therapy group than in the monotherapy group. Because there was no significant difference in terms of the emergence of antimicrobial resistance between the monotherapy and combination therapy groups, these findings suggest that empirical combination therapy may contribute to the reduction in mortality by accelerating eradication. Hence, the manner in which combination therapy decreases mortality in patients with *P. aeruginosa* infection is an interesting question. Unfortunately, we did not perform a prospective study that could evaluate this and future analyses of this type will be required to do so.

The present study has some limitations. First, it was retrospective and performed at a single center, so there is the potential for bias that would limit the ability to draw a firm conclusion about the direct impact of antimicrobial therapy. However, it has been shown that elaborate statistical methods, such as the marginal structural model using IPTW, provide a better method of control for confounding influences by improving the adjustment of differences between treatment groups [20]. Furthermore, we tried to evaluate the possible factors that could affect the clinical outcomes of patients with pneumonia as much as possible. Second, our study included only patients with bacteremia, so our finding may not be directly applicable to *Pseudomonas* pneumonia without bacteremia.

## Conclusions

In conclusion, our data strongly suggest that adequate empirical combination therapy can decrease mortality in patients with bacteremic *Pseudomonas* pneumonia. Additional prospective studies are therefore warranted.

## Competing interests

The authors declare that they have no competing interests.

## Authors’ contributions

Conceived and designed the study: SYP, SOL, SHC; Performed the study: SYP, HJP, SMM. Analyzed the data: SYP, KHP. YPC. Contributed reagents/materials/analysis tools: SYP, MNK, SHK, YSK, JHW. Wrote the paper: SYP, SHC. Critical review of the paper: All. All authors read and approved the final manuscript.

## Pre-publication history

The pre-publication history for this paper can be accessed here:

http://www.biomedcentral.com/1471-2334/12/308/prepub

## Supplementary Material

Additional file 1 **Table S1.** Logistic regression analysis of the risk factors for 28-day mortality in all patients. Click here for file

## References

[B1] Joo EJ Kang CI Ha YE Park SY Kang SJ Wi YM Lee NY Chung DR Peck KR Song JH Impact of inappropriate empiric antimicrobial therapy on outcome in Pseudomonas aeruginosa bacteraemia: a stratified analysis according to sites of infection Infection 2011 39 4 309 318 10.1007/s15010-011-0124-6 21594653

[B2] Fujitani S Sun HY Yu VL Weingarten JA Pneumonia due to Pseudomonas aeruginosa: part I: epidemiology, clinical diagnosis, and source Chest 2011 139 4 909 919 10.1378/chest.10-0166 21467058

[B3] National Nosocomial Infections Surveillance System National Nosocomial Infections Surveillance (NNIS) System Report, data summary from January 1992 through June 2004, issued October 2004 Am J Infect Control 2004 32 8 470 485 10.1016/j.ajic.2004.10.001 15573054

[B4] Sun HY Fujitani S Quintiliani R Yu VL Pneumonia due to Pseudomonas aeruginosa: part II: antimicrobial resistance, pharmacodynamic concepts, and antibiotic therapy Chest 2011 139 5 1172 1185 10.1378/chest.10-0167 21540216

[B5] El Solh AA Alhajhusain A Update on the treatment of Pseudomonas aeruginosa pneumonia J Antimicrob Chemother 2009 64 2 229 238 10.1093/jac/dkp201 19520717

[B6] Anderson ET Young LS Hewitt WL Antimicrobial synergism in the therapy of gram-negative rod bacteremia Chemotherapy 1978 24 1 45 54 10.1159/000237759 412648

[B7] Paul M Benuri-Silbiger I Soares-Weiser K Leibovici L Beta lactam monotherapy versus beta lactam-aminoglycoside combination therapy for sepsis in immunocompetent patients: systematic review and meta-analysis of randomized trials BMJ 2004 328 7441 668 681 10.1136/bmj.38028.520995.63 14996699 PMC381218

[B8] Safdar N Handelsman J Maki DG Does combination antimicrobial therapy reduce mortality in Gram-negative bacteraemia? A meta-analysis Lancet Infect Dis 2004 4 8 519 527 10.1016/S1473-3099(04)01108-9 15288826

[B9] Traugott KA Echevarria K Maxwell P Green K Lewis JS 2nd Monotherapy or combination therapy? The Pseudomonas aeruginosa conundrum Pharmacotherapy 2011 31 6 598 608 10.1592/phco.31.6.598 21923444

[B10] Heyland DK Dodek P Muscedere J Day A Cook D Canadian Critical Care Trials Group Randomized trial of combination versus monotherapy for the empiric treatment of suspected ventilator-associated pneumonia Crit Care Med 2008 36 3 737 744 10.1097/01.CCM.0B013E31816203D6 18091545

[B11] Chamot E Boffi El Amari E Rohner P Van Delden C Effectiveness of combination antimicrobial therapy for Pseudomonas aeruginosa bacteremia Antimicrob Agents Chemother 2003 47 9 2756 2764 10.1128/AAC.47.9.2756-2764.2003 12936970 PMC182644

[B12] Carratalà J Mykietiuk A Fernández-Sabé N Suárez C Dorca J Verdaguer R Manresa F Gudiol F Health care-associated pneumonia requiring hospital admission: epidemiology, antibiotic therapy, and clinical outcomes Arch Intern Med 2007 167 13 1393 1399 10.1001/archinte.167.13.1393 17620533

[B13] Mandell LA Wunderink RG Anzueto A Bartlett JG Campbell GD Dean NC Dowell SF File TM Jr Musher DM Niederman MS Torres A Whitney CG Infectious Diseases Society of America; American Thoracic Society Infectious Diseases Society of America/American Thoracic Society consensus guidelines on the management of community-acquired pneumonia in adults Clin Infect Dis 2007 44 S27 S72 10.1086/511159 17278083 PMC7107997

[B14] Kollef MH Shorr A Tabak YP Gupta V Liu LZ Johannes RS Epidemiology and outcomes of health-care-associated pneumonia: results from a large US database of culture-positive pneumonia Chest 2005 128 6 3854 3862 10.1378/chest.128.6.3854 16354854

[B15] American Thoracic Society; Infectious Diseases Society of America Guidelines for the management of adults with hospital-acquired, ventilator-associated, and healthcare-associated pneumonia Am J Respir Crit Care Med 2005 171 4 388 416 15699079 10.1164/rccm.200405-644ST

[B16] Garnacho-Montero J Sa-Borges M Sole-Violan J Barcenilla F Escoresca-Ortega A Ochoa M Cayuela A Rello J Optimal management therapy for Pseudomonas aeruginosa ventilator-associated pneumonia: an observational, multicenter study comparing monotherapy with combination antibiotic therapy Crit Care Med 2007 35 8 1888 1895 10.1097/01.CCM.0000275389.31974.22 17581492

[B17] Clinical and Laboratory Standards Institute Performance standards for antimicrobial testing: seventeenth informational supplement. CLSI document M100-S17 2007 Clinical and Laboratory Standards Institute, Wayne, PA

[B18] Magiorakos AP Srinivasan A Carey RB Carmeli Y Falagas ME Giske CG Harbarth S Hindler JF Kahlmeter G Olsson-Liljequist B Paterson DL Rice LB Stelling J Struelens MJ Vatopoulos A Weber JT Monnet DL Multidrug-resistant, extensively drug-resistant and pandrug-resistant bacteria: an international expert proposal for interim standard definitions for acquired resistance Clin Microbiol Infect 2012 18 3 268 281 10.1111/j.1469-0691.2011.03570.x 21793988

[B19] Robins JM Hernán MA Brumback B Marginal structural models and causal inference in epidemiology Epidemiology 2000 11 5 550 560 10.1097/00001648-200009000-00011 10955408

[B20] Suarez D Haro JM Novick D Ochoa S Marginal structural models might overcome confounding when analyzing multiple treatment effects in observational studies J Clin Epidemiol 2008 61 6 525 530 10.1016/j.jclinepi.2007.11.007 18471655

