# Supplementary material for: Impact of adequate empirical combination therapy on mortality from bacteremic Pseudomonas aeruginosa pneumonia
Source: BMC Infect Dis. 2012 Nov 16;12:308. doi: 10.1186/1471-2334-12-308 (PMC3519646; doi:10.1186/1471-2334-12-308)
Supplement: Additional file 1 — Table S1. Logistic regression analysis of the risk factors for 28-day mortality in all patients. [file 1471-2334-12-308-S1.doc]

Supplemental Table 1. Logistic regression analysis of the risk factors for 28-day mortality in all patients

|  | Univariate analysis |  | Multivariate analysis |  |
| --- | --- | --- | --- | --- |
| Variable | OR (95% CI) | *P* | OR (95% CI) | *P* |
| Age | 1.01 (0.98-1.04) | .48 |  |  |
| Male gender | 0.62 (0.25-1.54) | .30 |  |  |
| Adequacy of empirical therapy |  |  |  |  |
| Inadequate | 3.07 (1.29-7.31) | 0.01 | 2.73 (1.11-6.71) | 0.03 |
| Adequate | 1.0 (referent) |  | 1.0 (referent) |  |
| McCabe score |  |  |  |  |
| Non-fatal | 1.0 (referent) |  |  |  |
| Ultimately fatal | 1.10 (0.47-2.56) | .83 |  |  |
| Rapidly fatal | 1.98 (0.57-6.91) | .28 |  |  |
| APACHE II score | 1.09 (1.03-1.15) | .01 | 1.08 (1.02-1.15) | 0.01 |
| Pitt bacteremia score | 1.23 (1.04-1.46) | .02 |  |  |
| CPIS | 1.00 (0.74-1.35) | .99 |  |  |
| Type of pneumonia |  |  |  |  |
| Community-acquired | 1.0 (referent) |  |  |  |
| Healthcare-associated | 1.41 (0.38-5.23) | .60 |  |  |
| Hospital- acquired | 1.89 (0.62-5.77) | .27 |  |  |
| Ventilator-associated | 2.16 (0.58-8.04) | .25 |  |  |
| Without MDR-*P. aeruginosa* | 0.94 (0.37-2.39) | .90 |  |  |
| Without previous antibiotic therapy | 0.44 (0.20-0.99) | .05 |  |  |
| Initial manifestation within 24 h |  |  |  |  |
| Sepsis | 0.44 (0.17-1.13) | .09 |  |  |
| Severe sepsis | 0.47 (0.16-1.40) | .09 |  |  |
| Septic shock | 1.0 (referent) |  |  |  |

MDR = multidrug-resistant.
